# CMV Serostatus of Donor-Recipient Pairs Influences the Risk of CMV Infection/Reactivation in HSCT Patients

**DOI:** 10.1155/2012/375075

**Published:** 2012-11-22

**Authors:** Emilia Jaskula, Jolanta Bochenska, Edyta Kocwin, Agnieszka Tarnowska, Andrzej Lange

**Affiliations:** ^1^Department of Clinical Immunology, L. Hirszfeld Institute of Immunology and Experimental Therapy, Polish Academy of Sciences, 12 Rudolfa Weigla Street, 53-114 Wroclaw, Poland; ^2^Lower Silesian Center for Cellular Transplantation, National Bone Marrow Donor Registry, Grabiszyńska 105, 53-439 Wroclaw, Poland

## Abstract

CMV donor/recipient serostatus was analyzed in 200 patients allografted in our institution from unrelated (122 patients) donors and 78 sibling donors in the years 2002–2011 in relation to posttransplant complications. On a group basis independently of the CMV serostatus of donor-recipient pairs sibling transplantations and those from unrelated donors that matched 10/10 at allele level had a similar rate of CMV reactivation (17/78 versus 19/71, *P* = ns). The rate of CMV reactivation/infection was higher in patients grafted from donors accepted at the lower level of matching than 10/10 (18/38 versus 36/149, *P* = 0.008). The incidence of aGvHD followed frequencies of CMV reactivation in the tested groups, being 40/156 and 25/44 in patients grafted from sibling or unrelated donors that 10/10 matched and in those grafted from donors taht HLA mismatched, respectively (*P* = 0.001). Regarding the rate of reactivation in both groups seropositive patients receiving a transplant from seronegative donors had more frequently CMV reactivation as compared to those with another donor-recipient matching CMV serostatus constellation (22/43 versus 32/143, *P* = 0 < 0.001). Multivariate analysis revealed that seropositivity of recipients with concomitant seronegativity of donors plays an independent role in the CMV reactivation/infection (OR = 2.669, *P* = 0.037; OR = 5.322, *P* = 0.078; OR = 23.034, *P* = 0.023 for optimally matched and mismatched patients and the whole group of patients, resp.).

## 1. Introduction

Donor-recipient matching for unrelated hematopoietic stem cell transplantation (HSCT) in addition to human leukocyte antigens (HLA) includes CMV serostatus of the donor and recipient to facilitate the decision [1, 2].

In the clinical practice the presence of CMV IgM antibodies is suggestive of the active infection/reactivation and the presence of IgG antibodies indicates prior infection and shows CMV immunological competence of individuals [3–5]. Unfortunately, it is very suggestive that IgG CMV antibody positive individuals harbor CMV in a latent form and their blood products are infective for CMV incompetent recipients [6]. In the present era of specific anti-CMV chemotherapy the significant impact of pretransplant donor seropositivity on the patient outcome is controversial—reviewed in the Boeckh and Nichols paper [7]. However, recipient CMV serostatus still remains an important risk factor of the patient outcome [8, 9]. 

HSCT involving pairs in which both donor and recipient lack CMV IgG antibodies is associated with a lower transplant mortality [10]. In the latter situation we are dealing with a donor-recipient pair in which probably neither donor nor recipient has CMV in alatent form. On the other hand, positivity of both donor and recipient should also favor the HSCT outcome—both donors and recipients likely have CMV in a latent form but the immune system of the donor should have a memory of CMV infection, which facilitates the immune response to CMV posttransplant. However, Ljungman et al. [11] in the megafile analysis showed that the latter important observation seems to be valid only for the unrelated donor transplantation setting. 

To add new information to the still disputable association between the CMV donor/recipient serostatus with the outcome of transplantation the present study was undertaken.

## 2. Materials and Methods

Two hundred patients (F/M: 91/109; 26 and 174 patients were below and above 16 years of age, resp.) allografted from unrelated donors (122 patients), and 78 from sibling (SIB) donors in our institution in the years 2002–2011 were studied. One hundred and seventy-five suffered from hematological malignancies acute myeloid leukemia (AML; *n* = 67), chronic myeloid leukemia (CML; *n* = 24), acute lymphocytic leukemia (ALL; *n* = 39), other lymphoproliferative disorders (*n* = 23), myeloproliferative diseases (*n* = 10), and myelodysplastic syndromes (*n* = 12). The others were transplanted because of anemias (10 patients) and immunodeficiencies (14 patients) and osteopetrosis (*n* = 1).

One hundred and five and 95 patients received myeloablative (based on busulfan and cyclophosphamide) and reduced intensity conditioning (reduced busulfan dose or melphalan plus fludarabine and antithymocyte globulin (ATG)), respectively. Unrelated donor transplanted patients and those on reduced intensity conditioning received ATG (10 to 12.5 mg/kg b.w. cumulative dose, 125 patients) or alemtuzumab (90 mg as a dose, 38 patients) as a part of the conditioning regimen. All patients were on cyclosporin A with a dose adjusted to the blood CsA trough a level to 200 ng/L. CMV serostatus, age, gender, underlying disease, donor source, and HLA match as well as conditioning regimen (reduced or myeloablative) are given in Table 1.

The patients were routinely followed for clinical outcome in one-week intervals until 30 days posttransplant, then monthly until one year post-transplant and as well as when clinical symptoms were suggestive of CMV, EBV, or HHV6 reactivation or any other serious post-transplant complications including relapse or GvHD. Out of 200 patients studied viral CMV, EBV and HHV6 DNA copies in blood were determined in 187 recipients transplanted after the year 2003.

The Zeus Scientific, Inc. (NJ, USA), IgG and IgM ELISA test system was used for qualitative detection of CMV-specific antibodies in donors' and recipients' plasma. The ELISA kit was used according to the manufacturer's instructions. Briefly, microtiter plates, precoated with inactivated CMV antigen, were incubated with the recipient or donor plasma. Bound IgG or IgM was detected with peroxidase labeled anti-IgG and anti-IgM antibodies by the addition of the color substrate and reading by spectrometry. Results were interpreted as seropositive or seronegative as per the manufacturer's instructions.

DNA was extracted from peripheral blood (QiAmp Blood Kit; Qiagen, Hilden, Germany) according to the manufacturer's instructions. The numbers of CMV, EBV, and HHV6 DNA copies in peripheral blood cells were determined using real-time PCR and Light Cycler II (Roche, Mannheim, Germany). The sequences of the PCR primers and the probe were selected from the *BALF5* region of EBV, the *US17* region of CMV, and the *U67* region of HHV6. PCRs were performed as described by Jaskula et al. [12]. 

### 2.1. Statistical Analysis

Statistical analysis was performed using the CSS Statistica for Windows (version 10.0) software (Stat-Soft Inc., Tulsa, OK). Univariate analyses were performed by the Fisher exact test. Logistic regression was used for the multivariate analysis, and alog-rank test to analyze the survival probability. Differences between samples were considered to be significant when *P* < 0.05 and those between 0.05 and 0.1 were indicative of a trend.

## 3. Results and Discussion

**Figure 1:**
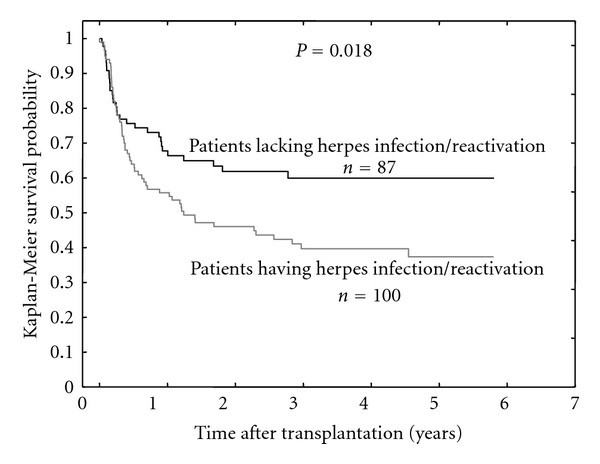
Overall survival in the groups of patients having and lacking *herpes* virus (CMV and/or EBV and/or HHV6) reactivations/infections.

The presence of >100 CMV, EBV, and HHV6 DNA copies per 10^5^ blood cells (clinically significant [12, 13]) was detected in 29%, 24%, and 18% of patients, respectively. Sixty out of 100 patients having during the observation period one or more reactivation events of one or more examined herpes viruses died. The mortality rate was lower in the group of patients lacking reactivations/infections (32 out of 87 patients), which resulted in a better five-year survival (59% versus 37%,  *P* = 0.018, Figure 1).

**Figure 2:**
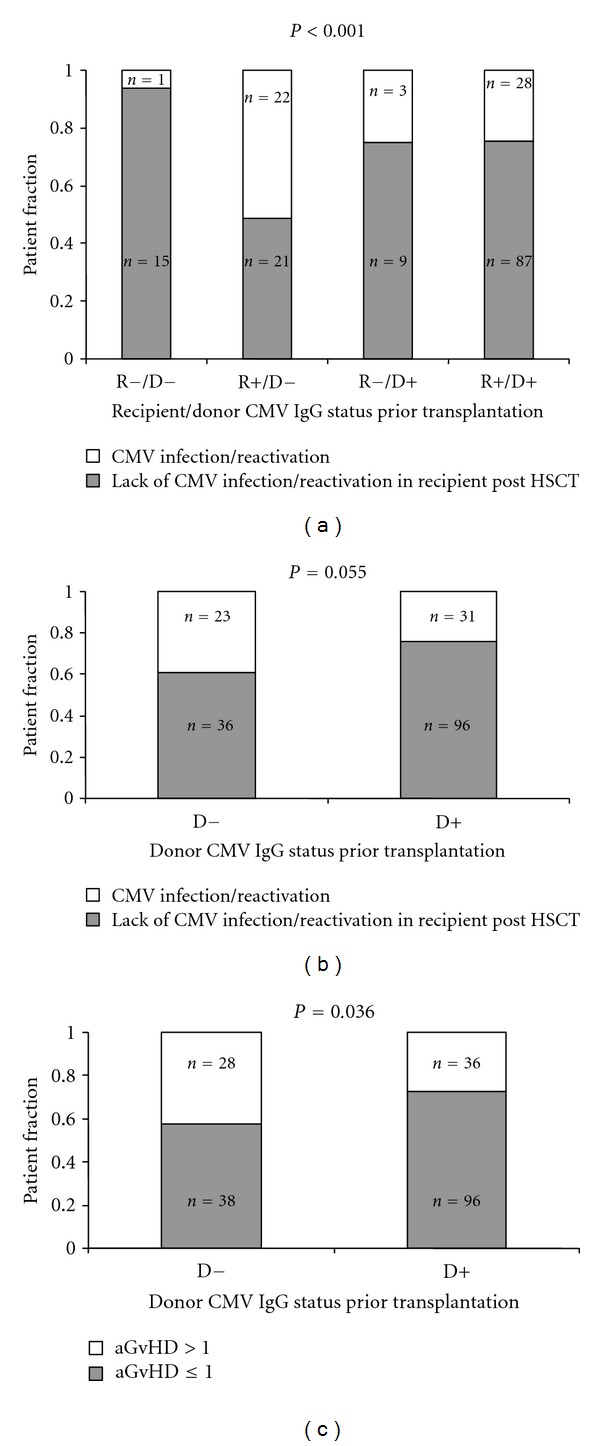
CMV reactivation/infection with respect to donor/recipient CMV serology (a) and donor CMV IgG status independently of the serostatus of recipients (b). Acute GvHD in patients transplanted from CMV IgG negative and CMV IgG positive donors (c) (R: recipient, D: donor, “+”: CMV IgG positive, and “−”: CMV IgG negative).

**Figure 3:**
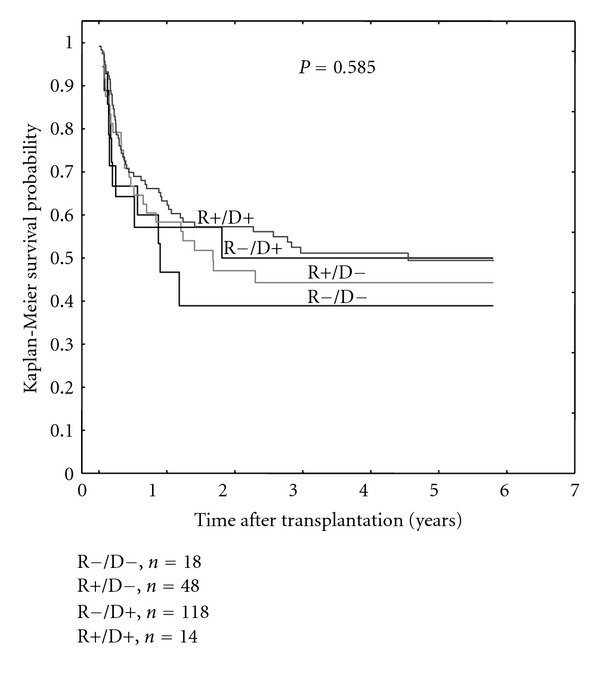
Survival of HSCT patients in the groups stratified according to CMV donor-recipient serostatus constellation (R: recipient, D: donor, “+”: CMV IgG positive, and “−”: CMV IgG negative).

Patients receiving transplantation from the CMV IgG seronegative donors tended to suffer more frequently from CMV infection/reactivation after HSCT as compared to those grafted from CMV seropositive donors (23/59 versus 31/127, *P* = 0.055, Figure 2(b)). This association was valid for seropositive and seronegative recipients. However, the highest risk of CMV reactivation was when seropositive recipients were transplanted from the seronegative donors (22/43 versus 32/143, *P* < 0.001Figure 2(a)). In contrast, CMV negative serostatus of both the donor and the recipient was associated with the lowest rate of the CMV reactivation (1 out of 16 patients) as compared to other recipient (R)/donor (D) CMV IgG serostatus relations, being 22/43 versus 28/115 versus 3/12, (*P* < 0.001) for R+/D−, R+/D+, and R−/D+, resp. (Figure 2(a)). We also found that aGvHD (grade > I) was more frequently seen in patients receiving grafts from IgG negative donors (28/66 versus 36/132, *P* = 0.036, Figure 2(c)). However, donor serostatus did not affect the survival of HSCT recipients (Figure 3).

In addition to the factors associated with the serostatus of donors and recipients, a lack of optimal donor/recipient HLA matching was associated with a higher risk of grade > I aGvHD (25/44 versus 40/156, *P* < 0.001) and with a higher rate of CMV reactivation/infection (18/38 versus 36/149, *P* = 0.008). CMV reactivation was also more frequently seen in patients who were over 16 years old at the time of transplantation (52/165 versus 2/22, *P* = 0.042, Table 2) and in those having CMV IgG antibodies before transplantation (50/159 versus 4/28, *P* = 0.073, Table 2). 

Multivariate analysis devoted to the evaluation of the risk factors of aGvHD showed that unrelated donor (OR = 2.591, *P* = 0.036) transplantation and HLA mismatch (OR = 2.361, *P* = 0.042) appeared as independent and significant factors associated with aGvHD grade > I (Table 3). In spite of the univariate results multivariate analysis did not confirm the role of CMV reactivation and donor serology as independent factors associated with aGvHD (Table 3). 

The next statistical approach was to validate factors associated with CMV reactivation. For that also a multivariate analysis was calculated employing factors as follows: recipient IgG serology, donor-recipient HLA mismatch, transplantation recipient in CMV IgG positive/donor CMV IgG negative serology, type of donors, recipient age, and aGvHD. Among the above factors donor-recipient HLA mismatch (OR = 3.499, *P* = 0.016), recipient CMV IgG positive/donor CMV IgG negative serology status constellation (OR = 23.030, *P* = 0.023), and recipient age over 16 years (OR = 9.865, *P* = 0.007) were found to be significant risk factors of CMV reactivation (Table 4). 

To further analyze the significance of CMV serology as a risk factor of CMV reactivation similar to that above, analysis was performed for groups consisting of SIB and MUD 10/10 matched and MUD not optimally matched (Tables 5 and 6). On agroup basis independently of the CMV serostatus of donor-recipient pairs, sibling transplantations and those from unrelated donors matched 10/10 at allele level had asimilar rate of CMV reactivation (17/78 versus 19/71, *P* = ns). Notably, the rate of CMV reactivation was higher in patients grafted from donors accepted at the lower level of matching than 10/10 (18/38 versus 36/149, *P* = 0.008). Also when we considered separately the optimal match group (SIB + MUD) the highest risk of CMV reactivation was observed when donors were negative but recipients were positive (12/28 versus 24/120, *P* = 0.015). In MUD HLA mismatched recipients a tendency to the association seropositivity of recipients with concomitant seronegativity of donors with the CMV reactivation/infection was observed (10/15 versus 8/23, *P* = 0.096). Notably in the MUD HLA mismatch group recipient age >16 years was a risk factor for CMV reactivation (17/28 versus 1/10, *P* = 0.009, Table 5). There were no significant associations between aGvHD and variables considered in this paper in the optimally matched group (SIB + 10/10 HLA matched) and in the MUD HLA mismatched group of patients. 

Multivariate analysis results of patients optimally matched and separately those not optimally matched were similar and revealed that among factors analyzed for the risk of CMV reactivation seropositivity of recipients with concomitant seronegativity of donors plays an independent role (OR = 2.670, *P* = 0.037) for optimally matched and as tendency in HLA mismatched patients (OR = 5.322, *P* = 0.078, Table 6). 

## 4. Conclusions

The information provided in the present paper shows that IgG negativity in donors favors the outcome of HSCT only when recipients are also CMV IgG negative. The worst is when donor IgG CMV negativity is confronting IgG CMV positivity in recipients. This confirms the importance of CMV IgG positivity likely associated with the immune competence of donors [3–5], which is of a special value in seropositive patients, very likely having CMV in alatent form [6]. Therefore, when recipients are IgG CMV positive the immune competence of donors is required to reduce the risk of CMV reactivation. This observation can be used as one of the factors that should be considered during donor selections for an optimal post-HSCT outcome. 

## Figures and Tables

**Table 1:** Patient characteristics.

Number of patients	200
Age	
(median, range), yrs	34, 1–60
Adults > 16 yrs	174
Children ≤ 16 yrs	26
Recipient gender	
Female	91
Male	109
Donor gender	
Female	83
Male	116
Donor	
Sibling	78
Unrelated HLA matched (10/10 at the allele level),	78
Mismatched, at the allele or low resolution levels up to two mismatches	44
Transplant material	
Bone marrow (BM)	28
Peripheral blood progenitor cells (PBPC)	172
Diagnosis	
Hematological malignancies (HM)	175
Chronic myeloid leukemia (CML)	24
Chronic lymphocytic leukemia (CLL)	5
Acute myeloid leukemia (AML)	67
Acute lymphocytic leukemia (ALL)	39
Other HM	40
Anemias and immunodeficiencies	24
Osteopetrosis	1
Conditioning regimen	
Myeloablative	105
Reduced intensity conditioning (RIC)	95
Acute GvHD, grades	
0	114
I	21
II	26
III	16
IV	23
Chronic GvHD	
Extensive	38
Limited	33
EBV ≥100 DNA copies/10^5^ cells	45/187
CMV ≥100 DNA copies/10^5^ cells	54/187
HHV6 ≥100 DNA copies/10^5^ cells	34/187
Polyoma (JC/BK)	19/33
CMV IgG serostatus	
Recipients	
CMV IgG negative	32
CMV IgG positive	168
Donors	
CMV IgG negative	66
CMV IgG positive	132
Recipient/donor CMV serostatus	
Recipient CMV IgG (+)/donor CMV IgG (+)	118
Recipient CMV IgG (−)/donor CMV IgG (−)	18
Recipient CMV IgG (+)/donor CMV IgG (−)	48
Recipient CMV IgG (−)/donor CMV IgG (+)	14

**Table 2:** Univariate analysis of risk factors for aGvHD and CMV reactivation/infection event(s) in patients post-alloHSCT.

Variable	aGvHD		*P* value	CMV absence	CMV presence	*P* value
	≤grade I	>grade I		Infection/reactivation until 1 year post-HSCT		
Donor/recipient HLA match						
Matched	116	40	***P ***<0.001	113	36	***P *** = 0.008
**Mismatched at the allele or low** **resolution levels up to two mismatches**	**19**	**25**		**20**	**18**	

**Source of HSCT**						
**PBPC**	**111**	**61**	***P *** = 0.029	112	48	*P* = 0.496
BM	24	4		21	6	

**Type of donor**						
SIB	65	13	***P ***<0.001	61	17	***P *** = 0.074
MUD	**70**	**52**		72	37	

Conditioning regimen						
RIC	67	28	*P* = 0.450	60	30	*P* = 0.202
Myeloablative	68	37		73	24	

**Donor CMV IgG**						
**CMV IgG−**	**38**	**28**	***P *** = 0.036	**36**	**23**	***P *** = 0.055
CMV IgG+	96	36		96	31	

**Recipient CMV IgG**						
CMV IgG−	19	13	*P* = 0.307	24	4	***P *** = 0.073
**CMV IgG+**	116	52		**109**	**50**	

**Donor-recipient IgG CMV serology**						
R−/D−	10	8		15	1	
R+/D−	28	20	*P* = 0.159	**21**	**22**	***P ***<0.001
R−/D+	9	5		9	3	
R+/D+	87	31		87	28	
R−/D−, R+/D−, R−/D+, R+/D+	106	44	*P* = 0.115	111	32	***P ***<0.001
**R+/D−**	28	20		**21**	**22**	

Donor/recipient gender						
Male to male, female to female, and male to female	105	54	*P* = 0.572	105	44	*P* = 0.841
Female to male	29	11		28	10	

Donor gender						
Male	76	40	*P* = 0.543	75	32	*P* = 0.747
Female	58	25		58	22	

Recipient gender						
Male	76	33	*P* = 0.544	71	27	*P* = 0.747
Female	59	32		62	27	

**Recipient age**						
≤16	17	9	*P* = 0.824	20	2	***P *** = 0.042
**>16**	118	56		**113**	**52**	

**CMV infection/reactivation event within 1 year post-HSCT**						
CMV−	97	36	***P *** = 0.025			
**CMV+**	**30**	**24**				

**aGvHD**						
aGvHD ≤ grade I				97	30	***P *** = 0.025
**aGvH**D > g**rade I**				**36**	**24**	

EBV infection/reactivation event within 1 year post HSCT						
EBV−	100	42	*P* = 0.204	104	38	*P* = 0.263
EBV+	27	18		29	16	

HHV6 infection/reactivation event within 1 year post HSCT						
HHV6−	103	50	*P* = 0.835	110	43	*P* = 0.677
HHV6+	24	10		23	11	

PBPC: peripheral blood progenitor cells; BM: bone marrow; R: recipient; D: donor; “−”: negative; “+”: positive; ATG: antithymocyte globulin; SIB: HLA-identical siblings; MUD: unrelated donors; RIC: reduced intensity conditioning.

**Table 3:** Multivariate analysis of risk factors for aGvHD (grade > I).

Variable	Coefficient	*P* value	Odds ratio	95% CI
CMV infection/reactivation event within 1 year post HSCT	0.5473	0.1362	1.7286	0.8415 to 3.5509
CMV IgG in donor serum	0.0290	0.9411	1.0295	0.4762 to 2.2254
**Donor-recipient HLA mismatch**	**0.8591**	**0.0421**	**2.3611**	**1.0310 to 5.4072**
**Unrelated donor**	**0.9520**	**0.0355**	**2.5909**	**1.0669 to 6.2921**
BM as a source of cells	−0.6177	0.3090	0.5392	0.1640 to 1.7723

**Table 4:** Multivariate analysis of risk factors for CMV reactivation/infection.

Variable	Coefficient	*P* value	Odds ratio	95% CI
Recipient CMV IgG seronegativity	−0.0761	0.9224	0.9267	0.2000 to 4.2929
**Donor/recipient HLA mismatch**	**1.2525**	**0.0155**	**3.4992**	**1.2695 to 9.6446**
**R CMV IgG+/D CMV IgG−**	**3.1370**	**0.0227**	**23.0340**	**1.5491 to 342.4999**
Unrelated donor	0.0021	0.9965	1.0021	0.3904 to 2.5722
aGvHD > 1	0.5363	0.1755	1.7096	0.7870 to 3.7141
**Recipient age **>16** years **	**2.2890**	**0.0072**	**9.8650**	**1.8606 to 52.3036**

**Table 5:** Univariate analysis of risk factors for CMV reactivation/infection event(s) in group of SIB and MUD HLA match patients and in group of MUD HLA mismatch patients.

	Optimally matched group (SIB+ 10/10 HLA matched) of patients			MUD HLA mismatched group of patients		
Variable	CMV absence	CMV presence	*P* value	CMV absence	CMV presence	*P* value
	Infection/reactivation until 1 year post HSCT			Infection/reactivation until 1 year post HSCT		
Source of HSCT						
PBPC	95	30	1.000	17	18	0.232
BM	18	6		3	0	

**Conditioning regimen**						
Absence of ATG and Campath	33	5	0.167			0.170
ATG	60	22		19	14	
Campath	20	9		1	4	
**RIC**	**53**	**23**	**0.087**	7	7	1.000
Myeloablative	60	13		13	11	

Donor CMV IgG						
CMV IgG−	26	12	0.273	10	11	0.532
CMV IgG+	86	24		10	7	

Recipient CMV IgG						
CMV IgG−	18	2	0.160	6	2	0.238
CMV IgG+	95	34		14	16	

**Donor-recipient IgG CMV serology**						
**R−/D−**	**10**	**0**		**5**	**1**	
R−/D+	8	2	**0.032**	1	1	**0.18**
**R+/D−**	**16**	**12**		**5**	**10**	
R+/D+	78	22		9	6	
R−/D−, R−/D+, R+/D+	96	24	**0.015**	15	8	**0.096**
**R+/D−**	**16**	**12**		**5**	**10**	

Donor/recipient gender						
Male to male, female to female, and male to female	67	23	0.698	13	14	0.485
Female to male	46	13		7	4	

Donor gender						
Male	67	23	0.569	8	9	0.746
Female	46	13		12	9	

Recipient gender						
Male	59	21	0.698	12	6	0.112
Female	54	15		8	12	

**Recipient age**						
≤16	11	1	0.290	9	1	**0.009**
>16	102	35		**11**	**17**	

aGvHD						
aGvHD ≤ grade I	88	23	0.123	9	7	0.752
aGvHD > grade I	25	13		11	11	

**Table 6:** Multivariate analysis of risk factors for CMV infection/reactivation in group of SIB and MUD HLA match patients and in group of MUD HLA mismatch patients.

Variable	Optimally matched group (SIB+ 10/10 HLA matched) of patients				MUD HLA mismatched group of patients			
	Coefficient	*P* value	Odds ratio	95% CI	Coefficient	*P* value	Oddsratio	95%CI
aGvHD > 1	0.7265	0.1015	2.0679	0.8667 to 4.9336	0.0185	0.9816	1.0187	0.2113 to 4.9106
Recipient CMV IgG seronegativity	0.9058	0.2601	2.474	0.5113 to 11.9711	0.5751	0.6126	1.7773	0.1918 to 16.4703
**R CMV IgG+/D CMV IgG−**	**0.9819**	**0.0374**	**2.6695**	**1.0587 to 6.7314**	**1.6719**	**0.0776**	**5.3222**	**0.8313 to 34.0733**
RIC conditioning regimen	−0.5655	0.1745	0.5681	0.2511 to 1.2849	0.30101	0.7333	1.3513	0.2391 to 7.6375
**Recipient age **>16** years**	1.2945	0.2318	3.6492	0.4371 to 30.4624	**3.1026**	**0.0148**	**22.256**	**1.8360 to 269.7835**

## References

[1] Ljungman P (2007). Risk assessment in haematopoietic stem cell transplantation: viral status. *Best Practice and Research*.

[2] International Standards for Cellular Therapy Product Collection (2008). *Processing, and Administration*.

[3] Gratama JW, Van Esser JWJ, Lamers CHJ (2001). Tetramer-based quantification of cytomegalovirus (CMV)-specific CD8^+^ T lymphocytes in T-cell-depleted stem cell grafts and after transplantation may identify patients at risk for progressive CMV infection. *Blood*.

[4] Maeker HT, Maino VC (2004). Analyzing T-cell responses to cytomegalovirus by cytokine flow cytometry. *Human Immunology*.

[5] Sester M, Gärtner BC, Sester U, Girndt M, Mueller-Lantzsch N, Köhler H (2003). Is the cytomegalovirus serologic status always accurate? A comparative analysis of humoral and cellular immunity. *Transplantation*.

[6] Roback JD (2002). CMV and blood transfusions. *Reviews in Medical Virology*.

[7] Boeckh M, Nichols WG (2004). The impact of cytomegalovirus serostatus of donor and recipient before hematopoietic stem cell transplantation in the era of antiviral prophylaxis and preemptive therapy. *Blood*.

[8] Nichols WG, Corey L, Gooley T, Davis C, Boeckh M (2002). High risk of death due to bacterial and fungal infection among cytomegalovirus (CMV)-seronegative recipients of stem cell transplants from seropositive donors: evidence for indirect effects of primary CMV infection. *Journal of Infectious Diseases*.

[9] McGlave PB, Shu XO, Wen W (2000). Unrelated donor marrow transplantation for chronic myelogenous leukemia: 9 years’ experience of the National Marrow Donor Program. *Blood*.

[10] Ljungman P, Larsson K, Kumlien G (2002). Leukocyte depleted, unscreened blood products give a low risk for CMV infection and disease in CMV seronegative allogeneic stem cell transplant recipients with seronegative stem cell donors. *Scandinavian Journal of Infectious Diseases*.

[11] Ljungman P, Brand R, Einsele H, Frassoni F, Niederwieser D, Cordonnier C (2003). Donor CMV serologic status and outcome of CMV-seropositive recipients after unrelated donor stem cell transplantation: an EBMT megafile analysis. *Blood*.

[12] Jaskula E, Dlubek D, Duda D, Bogunia-Kubik K, Mlynarczewska A, Lange A (2009). Interferon gamma 13-CA-repeat homozygous genotype and a low proportion of CD4^+^ lymphocytes are independent risk factors for cytomegalovirus reactivation with a high number of copies in hematopoietic stem cell transplantation recipients. *Biology of Blood and Marrow Transplantation*.

[13] Jaskula E, Dlubek D, Sedzimirska M, Duda D, Tarnowska A, Lange A (2010). Reactivations of cytomegalovirus, human herpes virus 6, and Epstein-Barr virus differ with respect to risk factors and clinical outcome after hematopoietic stem cell transplantation. *Transplantation Proceedings*.

